# Enhanced Electrochemical Performance of LiNi_0.5_Mn_1.5_O_4_ Composite Cathodes for Lithium-Ion Batteries by Selective Doping of K^+^/Cl^−^ and K^+^/F^−^

**DOI:** 10.3390/nano11092323

**Published:** 2021-09-07

**Authors:** Aijia Wei, Jinping Mu, Rui He, Xue Bai, Xiaohui Li, Lihui Zhang, Yanji Wang, Zhenfa Liu, Suning Wang

**Affiliations:** 1 School of Chemical Engineering and Technology, Hebei University of Technology, Tianjin 300130, China; weiaijia2012@126.com (A.W.); mu18822033042@sina.com (J.M.); yjwang@hebut.edu.cn (Y.W.); 2 Institute of Energy Resources, Hebei Academy of Sciences, Shijiazhuang 050081, China; kxyherui@163.com (R.H.); bx_ier@yeah.net (X.B.); nys2009@163.com (X.L.); zlhkxy@sohu.com (L.Z.); 3 Hebei Technology Innovation Center for Functional Material of Lithium Battery Electrolyte, Shijiazhuang 050081, China; 4 Guangxi Key Laboratory of Optical and Electronic Materials and Devices, College of Materials Science and Engineering, Guilin University of Technology, Guilin 541004, China; wangsuning0124@outlook.com

**Keywords:** LiNi_0.5_Mn_1.5_O_4_, K^+^/Cl^−^ co-doping, K^+^/F^−^ co-doping, rate capability, cycling stability

## Abstract

K^+^/Cl^−^ and K^+^/F^−^ co-doped LiNi_0.5_Mn_1.5_O_4_ (LNMO) materials were successfully synthesized via a solid-state method. Structural characterization revealed that both K^+^/Cl^−^ and K^+^/F^−^ co-doping reduced the Li*_x_*Ni_1__−_*_x_*O impurities and enlarged the lattice parameters compared to those of pure LNMO. Besides this, the K^+^/F^−^ co-doping decreased the Mn^3+^ ion content, which could inhibit the Jahn–Teller distortion and was beneficial to the cycling performance. Furthermore, both the K^+^/Cl^−^ and the K^+^/F^−^ co-doping reduced the particle size and made the particles more uniform. The K^+^/Cl^−^ co-doped particles possessed a similar octahedral structure to that of pure LNMO. In contrast, as the K^+^/F^−^ co-doping amount increased, the crystal structure became a truncated octahedral shape. The Li^+^ diffusion coefficient calculated from the CV tests showed that both K^+^/Cl^−^ and K^+^/F^−^ co-doping facilitated Li^+^ diffusion in the LNMO. The impedance tests showed that the charge transfer resistances were reduced by the co-doping. These results indicated that both the K^+^/Cl^−^ and the K^+^/F^−^ co-doping stabilized the crystal structures, facilitated Li^+^ diffusion, modified the particle morphologies, and increased the electrochemical kinetics. Benefiting from the unique advantages of the co-doping, the K^+^/Cl^−^ and K^+^/F^−^ co-doped samples exhibited improved rate and cycling performances. The K^+^/Cl^−^ co-doped Li_0.97_K_0.03_Ni_0.5_Mn_1.5_O_3.97_Cl_0.03_ (LNMO-KCl0.03) exhibited the best rate capability with discharge capacities of 116.1, 109.3, and 93.9 mAh g^−1^ at high C-rates of 5C, 7C, and 10C, respectively. Moreover, the K^+^/F^−^ co-doped Li_0.98_K_0.02_Ni_0.5_Mn_1.5_O_3.98_F_0.02_ (LNMO-KF0.02) delivered excellent cycling stability, maintaining 85.8% of its initial discharge capacity after circulation for 500 cycles at 5C. Therefore, the K^+^/Cl^−^ or K^+^/F^−^ co-doping strategy proposed herein will play a significant role in the further construction of other high-voltage cathodes for high-energy LIBs.

## 1. Introduction

Currently, high-energy-density lithium-ion batteries (LIBs) are in urgent demand due to their wide applications in plug-in hybrid electric vehicles, portable electronic devices, and renewable energy storage devices [1,2,3,4]. The electrochemical performances of LIBs are mainly dependent on the properties of both the cathode and anode materials, especially the cathode materials. The LiNi_0.5_Mn_1.5_O_4_ (LNMO) cathode, with a higher operating voltage (4.7 V) and theoretical capacity (147 mAh g^−1^), is favored for its high energy density. Furthermore, LNMO possesses a three-dimensional framework structure, and the cost of the raw materials is relatively low, making it suitable for large-scale applications [5]. However, LNMO suffers from the undesirable generation of Li*_x_*Ni_1__−_*_x_*O or NiO impurity phases and side reactions at the electrode interface at high potential, leading to structural instability and severe capacity fading during cycling [6].

To address the problems mentioned above, researchers have attempted to introduce metal cations or anions with similar radii into LNMO at different sites (Li, Ni, Mn, or O). Most of the previous studies were restricted to single-ion doping. For example, Li^+^ and Na^+^ ions have been incorporated into the Li site to synthesize Li_1+*x*_Ni_0.5_Mn_1.5_O_4_ and Li_1−*x*_Na*_x_*Ni_0.5_Mn_1.5_O_4_ materials [7,8,9]. For Ni and Mn sites, metal and non-metal cations, such as Al^3+^ [10], Cu^2+^ [11], Y^3+^ [12], Co^3+^ [13], Cr^3+^ [14], Zr^4+^ [15], P^5+^ [16], B^3+^ [17], Ti^4+^ [18], and V^5+^ [19], have been introduced by various synthesis methods. For the O site, F^−^ [20] and Cl^−^ [21] ions have been used to replace the oxygen. However, single doping may affect the LNMO’s electrochemical performance in certain aspects. Ion co-doping is an effective strategy to simultaneously stabilize the crystal structure and enhance the electrochemical properties of LNMO materials. Such materials include Al^3+^, Cr^3+^, and F^−^ co-doped LNMO [22]; Li^+^ and F^−^ co-doped LNMO [23,24]; Mg^2+^ and F^−^ co-doped LNMO [25]; Cu^2+^ and Al^3+^ co-doped LNMO [26,27]; Cu^2+^, Al^3+^, and Ti^4+^ co-doped LNMO [28]; Mg^2+^ and Si^4+^ co-doped LNMO [29]; and Ti^4+^ and La^3+^ co-doped LNMO [30]. Among these co-doped ions, there are few reports of cation and anion co-doping in LNMO. Cation and anion co-doping has a unique advantage in that both ions can play a synergistic role in the impact of LNMO on the structure and properties. This approach has also been widely applied to improve the rate capability and cycling stability of LNMO. Sha et al. prepared a multi-substituted LiNi_0.475_Al_0.01_Cr_0.04_Mn_1.475_O_3.95_F_0.05_ cathode through a sol–gel method. After a series of tests, the Al^3+^/Cr^3+^/F^−^ co-doped sample possessed excellent rate performance and cycling stability. Moreover, the co-doping also enhanced the cycling stability at room temperature (20 °C) and an elevated temperature (55 °C) [22]. In our previous work, Mg^2+^ and F^−^ ions were incorporated into LNMO. The Mg^2+^/F^−^ co-doping increased the quantities of Mn^3+^ ions and the lattice parameters. In addition, the Mg^2+^/F^−^ co-doping increased the particle size, which could reduce the number of side reactions to some extent. The electrochemical results showed that the Mg^2+^/F^−^ co-doped LNMO-MF sample obtained excellent rate performance and cycling stability at a high C-rate of 5C [25].

As K^+^ possesses a larger radius (0.133 nm) than Li^+^ (0.076 nm), it is often used as a doping ion to replace Li^+^ in several cathode materials, including LiMn_2_O_4_ [31], LiNi_0.5_Co_0.2_Mn_0.3_O_2_ [32], Li_1.2_Ni_0.2_Mn_0.6_O_2_ [33], and Li_1.2_Ni_0.13_Co_0.13_Mn_0.54_O_2_ [34]. The K^+^ doping decreases the cation mixing and expands the Li layer spacing, thereby enhancing the structural stability and accelerating the Li^+^ diffusion in the bulk lattice. As reported by Yang et al., K^+^-doped Li_1.2_Ni_0.2_Mn_0.6_O_2_ was prepared via a sol–gel method. The K^+^ doping stabilized the surface O^2−^ and reduced the Mn^3+^ ion content. The electrochemical results showed that the electrode capacity retention in K^+^-doped Li_1.2_Ni_0.2_Mn_0.6_O_2_ was 99.96% after 100 cycles, and it exhibited outstanding cycling stability [33]. F^−^ (328 kJ mol^−1^) and Cl^−^ (349 kJ mol^−1^) ions, with larger electron affinities, are commonly used elements for O^2−^ (141 kJ mol^−1^) site substitution in LNMO cathodes, and they can stabilize the crystal structure and reduce the Li*_x_*Ni_1__−_*_x_*O or NiO impurity generation due to the stronger Mn-F/Ni-F bonds or Mn-Cl/Ni-Cl bonds as compared to Mn-O/Ni-O bonds [21].

Based on the studies described above, investigations into K^+^/Cl^−^ or K^+^/F^−^ co-doping effects on the crystal structures and electrochemical performances of LNMO are still lacking. Hence, pure LNMO, K^+^/Cl^−^ co-doped Li_1−*x*_K*_x_*Ni_0.5_Mn_1.5_O_4−*x*_Cl*_x_* (*x* = 0.02 and 0.03), and K^+^/F^−^ co-doped Li_1−*x*_K*_x_*Ni_0.5_Mn_1.5_O_4−*x*_F*_x_* (*x* = 0.01 and 0.02) materials were prepared via a solid-state method. The characterization indicated that the influences of K^+^/Cl^−^ co-doping on the structures and morphologies were different from those of K^+^/F^−^ co-doping. The electrochemical results substantiated that both the K^+^/Cl^−^ and K^+^/F^−^ co-doped LNMO materials exhibited better rate and cycling performances than pure LNMO. The K^+^/Cl^−^ co-doping was more conducive to improvement in the rate properties of LNMO, while the K^+^/F^−^ co-doping tended to enhance the cycling stability. Hence, the structures, morphologies, and electrochemical performances of pure LNMO, K^+^/Cl^−^ co-doped Li_1−*x*_K*_x_*Ni_0.5_Mn_1.5_O_4−*x*_Cl*_x_*, and K^+^/F^−^ co-doped Li_1−*x*_K*_x_*Ni_0.5_Mn_1.5_O_4−*x*_F*_x_* were comprehensively compared in this study.

## 2. Experimental Section

### 2.1. Material Preparation

A simple solid-state ball-milling process followed by a high-temperature calcination procedure was used to synthesize pure LNMO, K^+^/Cl^−^ co-doped, and K^+^/F^−^ co-doped samples. The synthesis process was as follows. First, stoichiometric amounts of Li_2_CO_3_ (5% excess), NiO, MnO_2_, and KCl (0.525:0.5:1.5:*x* by mole) were dispersed in ethanol by ball-milling (Pulverisette 7, Fritsch, Ida, Germany) for 10 min at 200 rpm and then for 210 min at 400 rpm to generate a homogeneous mixture. The obtained mixture was dried at 100 °C for 3 h to evaporate the ethanol. Finally, the obtained powder was annealed at 500 °C for 250 min and 850 °C for 8 h in air to form the K^+^/Cl^−^ co-doped Li_1−*x*_K*_x_*Ni_1.5_Mn_0.5_O_4−*x*_Cl*_x_* (*x* = 0, 0.02, and 0.03) samples (henceforth referred to as pure LNMO, LNMO-KCl0.02, and LNMO-KCl0.03, respectively). The K^+^/F^−^ co-doped Li_1−*x*_K*_x_*Ni_1.5_Mn_0.5_O_4−*x*_F*_x_* (*x* = 0.01, 0.02) samples, using KF as the dopant, were obtained using the same synthesis process as that used for the Li_1−*x*_K*_x_*Ni_1.5_Mn_0.5_O_4−*x*_Cl*_x_* samples. The obtained samples were referred to as LNMO-KF0.01 and LNMO-KF0.02. The LNMO-KCl0.04, LNMO-KF0.005, and LNMO-KF0.03 samples shown in the Supporting Information were also prepared via the same synthesis method.

### 2.2. Material Characterization

The crystalline structures and lattice parameters of all samples were characterized by X-ray diffraction (XRD, Ultima IV, Rigaku, Tokyo, Japan) with Cu Kα radiation in the 2θ range of 10–90°. The Rietveld refinement was carried out through TOPAS 4.2 software. The functional groups of the as-prepared samples were obtained by examining their Raman spectra (Raman, RM2000, Renishaw, London, UK). The surface elemental states and distributions of the samples were investigated via X-ray photoelectron spectroscopy (XPS, ESCALAB 250Xi XPS, Thermo Fisher Scientific, San Jose, CA, USA) with λ = 633 nm. The material morphological characteristics were evaluated by scanning electron microscopy (SEM, SU 8020, Hitachi Limited, Tokyo, Japan) and transmission electron microscopy (TEM, JEM-2100 plus, JEOL, Tokyo, Japan). Energy-dispersive spectroscopy (EDS, APOLLO XL, EDAX, Mahwah, NJ, USA) mappings were used for surface element characterization of the co-doped LNMO samples.

### 2.3. Electrochemical Measurements

Electrochemical tests of all the samples were performed by assembling CR2032-type coin cells (Shenzhen Meisen Electromechanical Equipment Co., Ltd., Shenzhen, China). The positive electrodes were prepared by blending the co-doped LNMO powders, poly (vinylidene fluoride), and super-P carbon (8:1:1 by wt.%). The mixture was dispersed in an N-methyl-2-pyrrolidone solvent, and the resulting slurries were coated onto Al foil and then dried at 105 °C for 12 h under vacuum. The mass loading of the corresponding active materials was ≈1.5 mg cm^−2^ on each electrode. In an Ar-filled glovebox, the coin cells were assembled with metallic lithium as the negative electrode, a porous polypropylene separator (Celgard 2400, Charlotte Manufacturing Facility, Charlotte, NC, USA), and a carbonate-based electrolyte (1 M LiPF_6_/EC+EMC+DMC (1:1:1 by volume)). The charge and discharge measurements of the cells were carried out in the voltage range of 3.5−5.0 V (1C = 140 mA g^−1^) using a testing system (CT2001A, Land, Wuhan Shenglan Electronic Technology Co., Ltd., Wuhan, China) at 25 °C. Cyclic voltammetry (CV, 0.1−0.5 mV s^−1^) and electrochemical impedance spectroscopy (EIS, 100 kHz–0.01 Hz, Gamry, Progress, PA, USA) tests were conducted using an electrochemical workstation (Interface 1000, Gamry, PA, USA) between 3.5 and 5.0 V.

## 3. Results and Discussion

### 3.1. Material Characterization

**Figure 1:**
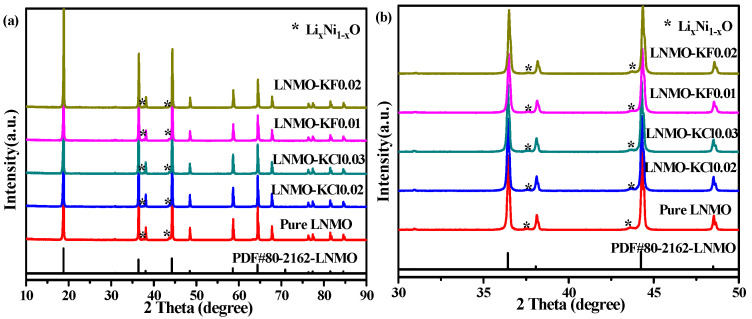
XRD patterns of all samples compared with the standard LNMO pattern (**a**); Enlarged region of 30–50 degrees for all samples (**b**).

The XRD results of the pure LNMO, LNMO-KCl0.02, LNMO-KCl0.03, LNMO-KF0.01, and LNMO-KF0.02 are shown in Figure 1a. The diffraction peaks of all the patterns were well indexed to the standard diffraction pattern of spinel structures (JCPDS No. 80–2162). The impurities (marked with *) in the five samples are Li*_x_*Ni_1__−_*_x_*O phases generated by LNMO losing oxygen when its calcination temperature exceeds 700 °C, as shown in Figure 1b. Based on the XRD patterns for all the samples, the Rietveld refinements were carried out (in Figure S1), and the Li*_x_*Ni_1__−_*_x_*O contents and lattice parameters are listed in Table 1. The contents of Li*_x_*Ni_1__−_*_x_*O for the pure LNMO, LNMO-KCl0.02, LNMO-KCl0.03, LNMO-KF0.01, and LNMO-KF0.02 samples were 3.9, 3.3, 1.5, 2.4, and 2.0 wt.%, respectively. The contents of Li*_x_*Ni_1__−_*_x_*O impurities were reduced by the K^+^/Cl^−^ and K^+^/F^−^ co-doping because Mn-Cl/Ni-Cl and Mn-F/Ni-F bonds are stronger than Mn-O/Ni-O bonds, which prevented oxygen release from the host lattice [21]. The calculated lattice parameters for the pure LNMO, LNMO-KCl0.02, LNMO-KCl0.03, LNMO-KF0.01, and LNMO-KF0.02 samples were 8.1726(8), 8.1771(0), 8.1815(2), 8.1768(6), and 8.1754(0), respectively. Compared to pure LNMO, the K^+^/Cl^−^ co-doping caused the lattice parameters to become larger as the K^+^/Cl^−^ co-doping content increased due to the larger ionic radius of K^+^ (0.133 nm) than that of Li^+^ (0.076 nm) and the larger ionic radius of Cl^−^ (0.181 nm) than that of O^2^^−^ (0.140 nm). This expanded the Li^+^ transport channels and facilitated Li^+^ diffusion [35]. LNMO-KF0.01 and LNMO-KF0.02 both possessed larger lattice parameters than pure LNMO. However, as the K^+^/F^−^ co-doping content increased, the lattice parameters of LNMO-KF0.005 (8.17508), LNMO-KF0.01 (8.17686), LNMO-KF0.02 (8.17540), and LNMO-KF0.03 (8.16980) increased first and then decreased (Figure S2 shows the Rietveld refinements of LNMO-KF0.005 and LNMO-KF0.03). The reason for this phenomenon was mainly that the K^+^/F^−^ co-doping caused the LNMO-KF0.02 and LNMO-KF0.03 to become more ordered structures with truncated octahedral shapes and the Mn^3+^ ion content to decrease; this was verified by further characterizations, as discussed below.

**Figure 2:**
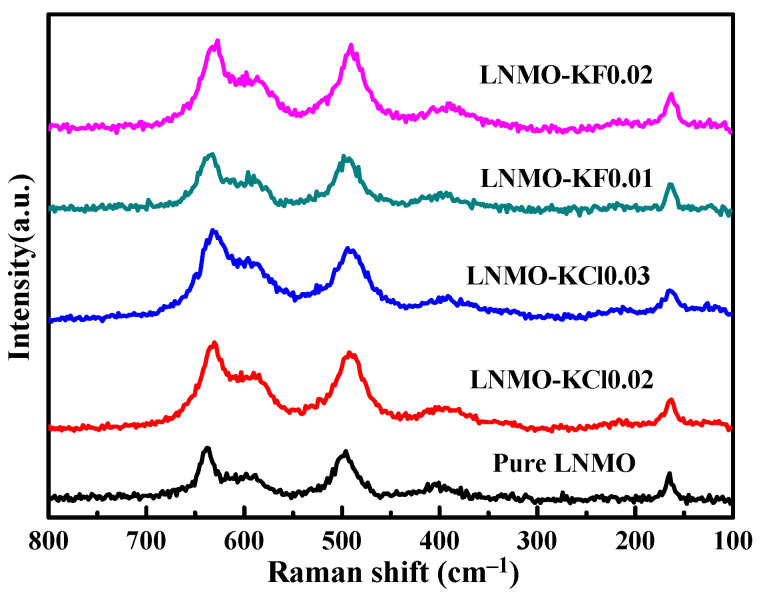
Raman spectra of all the samples.

Distinguishing the crystal structures of all the samples required the use of Raman spectroscopy, and the results indicated that they possessed disordered *Fd-*3m or ordered *P4_3_32* space groups, as shown in Figure 2. All the samples exhibited intense peaks in the ~636 cm^−1^ region, ascribed to the symmetric Mn-O stretching mode of the MnO_6_ groups, and two other peaks in the ~400 and 495 cm^−1^ region, attributed to the Ni^2+^-O stretching mode [36]. The peak intensity at 636 cm^−1^ was much stronger than that at 596 cm^−1^, demonstrating that all the samples are characteristic of typical disordered *Fd*-3m structures, based on a previous report [37]. Moreover, the splitting peaks between 580 and 630 cm^−1^ and the relatively higher peak intensity around 164 cm^−1^ of the LNMO material are typically ascribed to the ordered *P4_3_32* structure [37]. The LNMO-KCl0.02 and LNMO-KCl0.03 samples produced similar peaks to the pure LNMO, suggesting that the K^+^/Cl^−^ co-doping did not affect the crystal structures. However, upon increasing the K^+^/F^−^ co-doping content, slight peak splitting occurred at around 580–600 cm^−^^1^, and peaks with slightly higher intensity appeared at around 164 cm^−1^ for the LNMO-KF0.01 and LNMO-KF0.02, indicating that the crystal structure tended to show enhanced cation ordered degree by K^+^/F^−^ co-doping.

**Figure 3:**
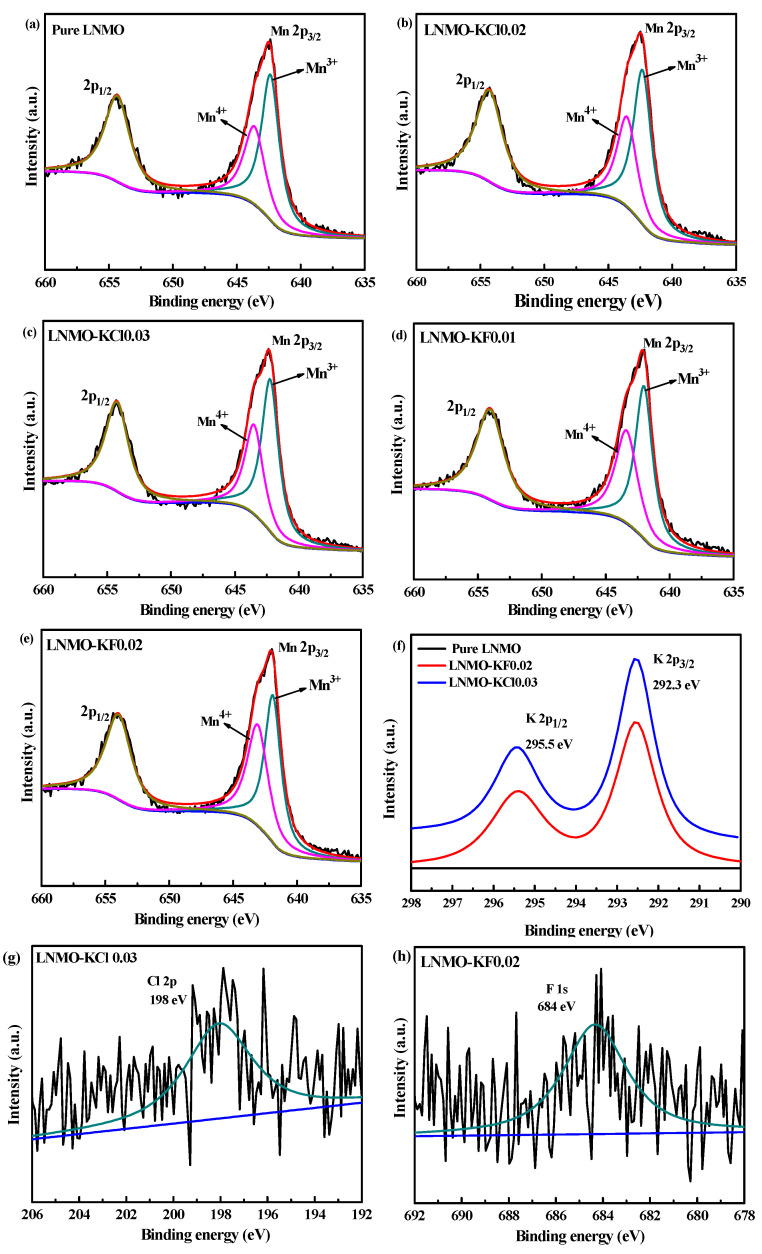
XPS spectra of Mn 2p regions for pure LNMO (**a**), LNMO-KCl0.02 (**b**), LNMO-KCl0.03 (**c**), LNMO-KF0.01 (**d**), and LNMO-KF0.02 (**e**); XPS spectra of K 2p for LNMO-KCl0.03 and LNMO-KF0.02 (**f**); XPS spectrum of Cl 2p for LNMO-KCl0.03 (**g**); XPS spectrum of F 1s for LNMO-KF0.02 (**h**).

To confirm the detailed surface oxidation states of the elements in pure LNMO and the co-doped samples, XPS was performed, and the corresponding spectra are shown in Figure 3. The Mn 2p spectra of the five samples consisted of two main peaks at ~654 and ~642 eV, assigned to Mn 2p_1/2_ and Mn 2p_3/2_, respectively, which matched well with the valence state of Mn^4+^ [38], as presented in Figure 3a–e. The peaks of Mn 2p_3/2_ fitted by the XPSPEAK software indicated the existence of a mixture of Mn^3+^ (642.3 eV) and Mn^4+^ (643.4 eV). The proportions of Mn^3+^ calculated based on the peak areas were 61.4%, 61.3%, 61.4%, 56.5%, and 53.5% for pure LNMO, LNMO-KCl0.02, LNMO-KCl0.03, LNMO-KF0.01, and LNMO-KF0.02, respectively. The K^+^/Cl^−^ co-doping did not affect the amount of Mn^3+^. However, the content of Mn^3+^ was inclined to decrease with increasing K^+^/F^−^ co-doping content, which agrees with the CV and the charge–discharge curves at the 4 V plateau discussed below. The Mn^3+^ contents of the five samples calculated by XPS were much higher than those calculated at the 4 V plateau during the charge–discharge tests, which may have been due to the existence of more Mn^3+^ on the surfaces of the samples instead of inside the structures [39,40]. Figure 3f displays the K 2p XPS spectra of LNMO-KCl0.03 and LNMO-KF0.02. A corresponding satellite peak at ~295.0 eV (assigned to K 2p_1/2_) is evident in both samples after a major peak at ~292.0 eV (ascribed to K 2p_3/2_). This is consistent with the characteristics of the valence state of K^+^ [32]. For the LNMO-KCl0.03 and LNMO-KF0.02 samples, the Cl 2p_3/2_ (Figure 3g) and F 1s (Figure 3h) peaks were located at 198.2 and 684.3 eV, respectively, indicating that the chemical valences of the Cl and F ions were both −1 in the two samples [20,41].

**Figure 4:**
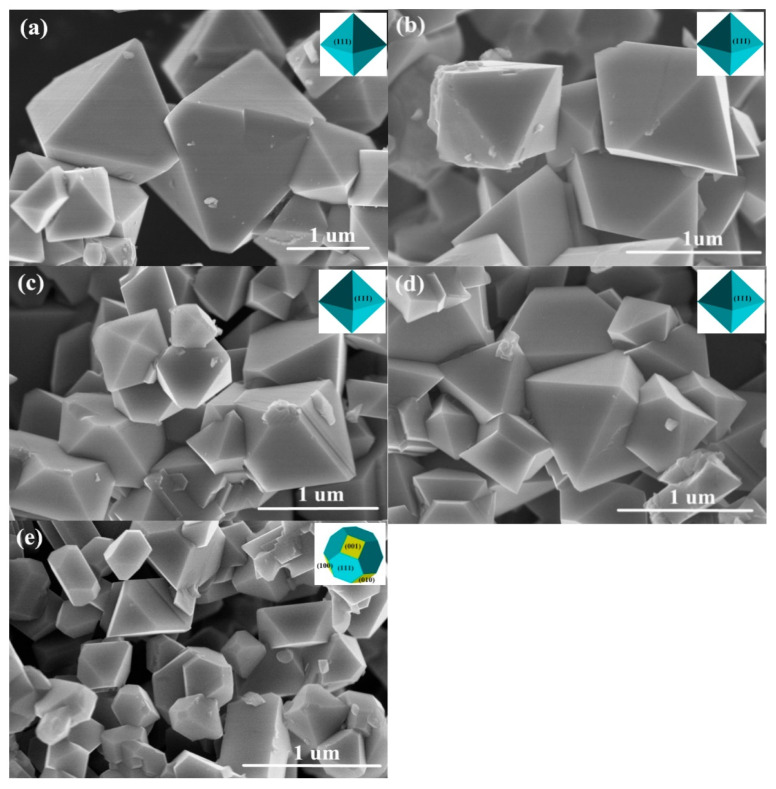
SEM images of pure LNMO (**a**), LNMO-KCl0.02 (**b**), LNMO-KCl0.03 (**c**), LNMO-KF0.01 (**d**), and LNMO-KF0.02 (**e**).

**Figure 5:**
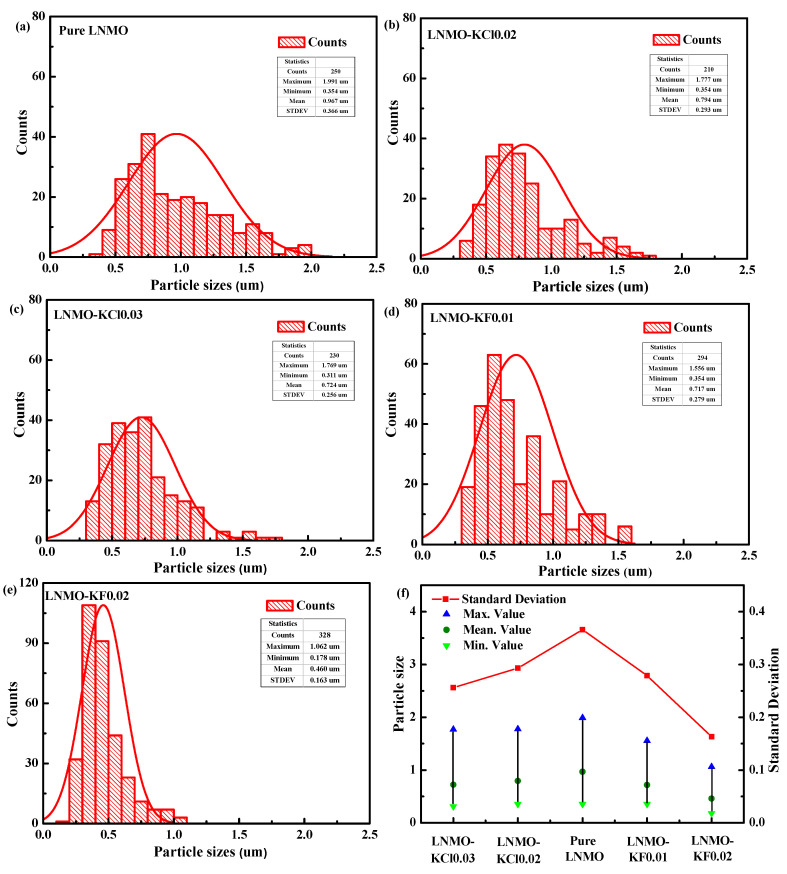
Particle size distributions of pure LNMO (**a**), LNMO-KCl0.02 (**b**), LNMO-KCl0.03 (**c**), LNMO-KF0.01 (**d**), and LNMO-KF0.02 (**e**); The standard deviations for the five samples (**f**).

Figure 4 shows the particle morphologies of the five samples. The pure LNMO exhibited mainly octahedral shapes with (111) crystal faces, shown in Figure 4a. LNMO-KCl0.02 and LNMO-KCl0.03 also exhibited octahedral morphologies (Figure 4b,c). However, the morphologies of K^+^/F^−^ co-doped particles were different from those of the K^+^/Cl^−^ co-doped particles. As the K^+^/F^−^ co-doping content increased, the LNMO-KF0.02 showed a truncated octahedral structure with mainly a {111} crystal face and extra {100} faces, as shown in Figure 4d,e. The positive {100} faces facilitated Li^+^ diffusion in the LNMO-KF0.02 sample, which was beneficial for its electrochemical performance [42,43]. To further investigate the effect of K^+^/Cl^−^ and K^+^/F^−^ co-doping on the particle size, the particle size distributions were determined for the five samples (about 200–250 particles for each sample in Figure S3), as depicted in Figure 5a–e. The particle sizes of both the K^+^/Cl^−^ and the K^+^/F^−^ co-doped samples decreased with increasing co-doping amount. Compared with the particle size of pure LNMO (0.967 µm), the particle sizes of LNMO-KCl0.02, LNMO-KCl0.03, LNMO-KF0.01, and LNMO-KF0.02 were smaller at 0.794, 0.724, 0.717 and 0.460 µm, respectively. The K^+^/Cl^−^ and K^+^/F^−^ co-doping both narrowed the particle size distribution, and LNMO-KF0.02 exhibited the smallest particle size. Figure 5f shows that the standard deviations of the K^+^/Cl^−^ or K^+^/F^−^ co-doped samples were lower than that of the pure LNMO, and LNMO-KF0.02 had the smallest standard deviation, suggesting that the K^+^/Cl^−^ and K^+^/F^−^ co-doping resulted in a more uniform particle distribution [44]. The smaller and more homogeneous particle sizes could shorten the Li^+^ diffusion length. However, the larger surface area of the co-doped samples could accelerate the side reactions between the cathode and electrolyte, leading to capacity fading [16]. Figures S4 and S5 present the EDS mappings of the LNMO-KCl0.03 and LNMO-KF0.02 samples, respectively. O, Mn, and Ni elements were distributed in the two samples. Moreover, K/Cl and K/F were also dispersed homogeneously in the LNMO-KCl0.03 and LNMO-KF0.02 samples, respectively, and were incorporated uniformly.

**Figure 6:**
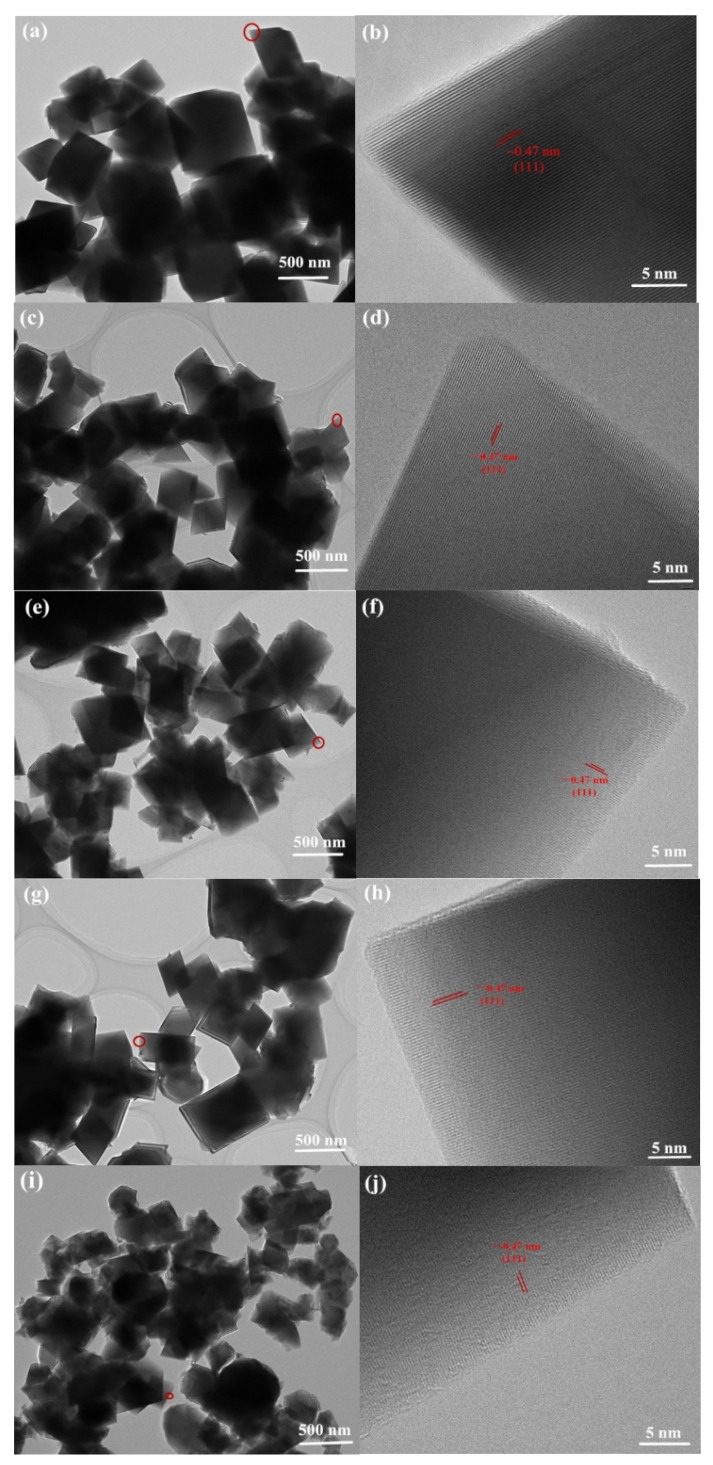
TEM and HR-TEM images of pure LNMO (**a**,**b**), LNMO-KCl0.02 (**c**,**d**), LNMO-KCl0.03 (**e**,**f**), LNMO-KF0.01 (**g**,**h**), and LNMO-KF0.02 (**i**,**j**).

Figure 6a–j shows the TEM and high-resolution TEM (HR-TEM) images of all the samples, which were used to gain more insight into the morphology changes. The K^+^/Cl^−^ co-doped LNMO-KCl0.02 and LNMO-KCl0.03 samples possessed the same octahedral structures as the pure LNMO, and the particle sizes of the LNMO-KCl0.02 and LNMO-KCl0.03 were slightly smaller than those of the pure LNMO. In addition, Figure 6b,d,f shows clear lattice fringes with d-spacing of ~0.47 nm for the pure LNMO, LNMO-KCl0.02, and LNMO-KCl0.03, which matched well with the interplanar distance of the (111) spaces [25]. The K^+^/F^−^ co-doped LNMO-KF0.01 and LNMO-KF0.02 samples also possessed smaller particle sizes compared to the pure LNMO (Figure 6g,i). The lattice fringes measured at ~0.47 nm were obtained along the (111) planes shown in Figure 6h,j. The LNMO-KF0.02 also exhibited a truncated octahedral morphology, consistent with SEM results.

### 3.2. Electrochemical Properties

**Figure 7:**
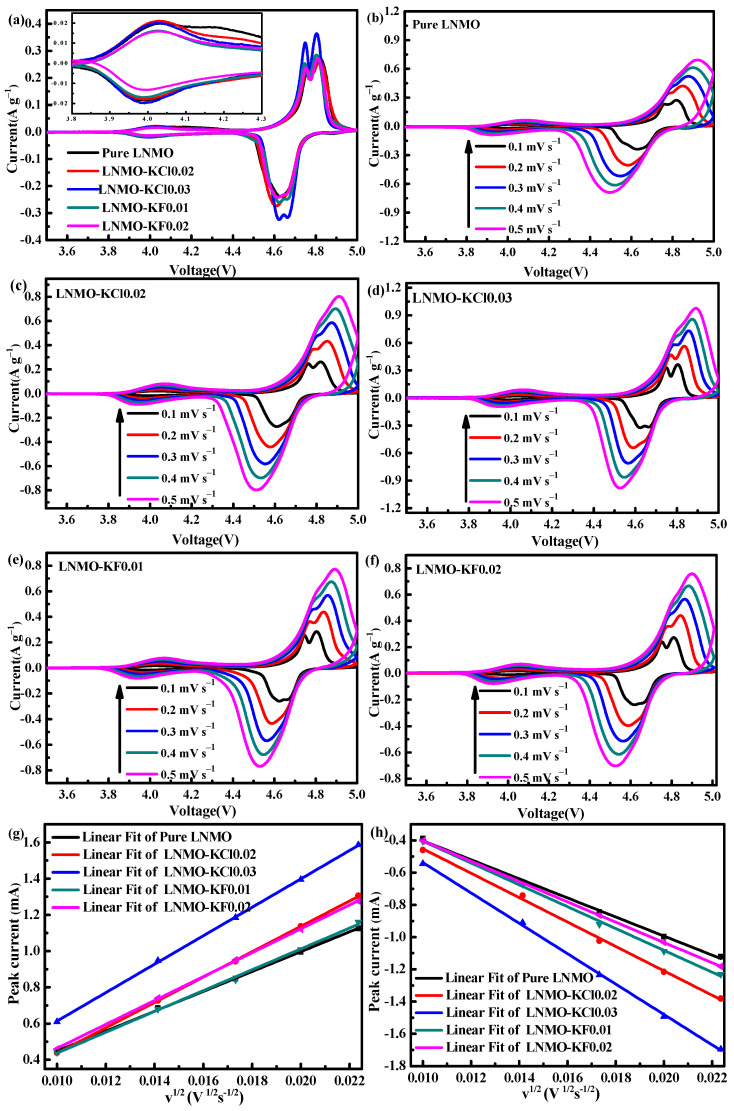
Cyclic voltammetry curves for all samples at 0.1 mV s^−1^ (**a**) and for samples individually at scan rates from 0.1 mV s^−1^ to 0.5 mV s^−1^ (**b**–**f**); Plots of the peak current (*i*_p_) vs. the square root of the scan rate (*v*^1/2^) (**g**,**h**).

To investigate the effects of K^+^/Cl^−^ and K^+^/F^−^ co-doping on the electrochemical kinetics of Li^+^ intercalation/deintercalation in LNMO, CV tests for the electrodes were performed in the voltage range of 3.5–5.0 V. Figure 7a shows the CV curves of all samples at a scan rate of 0.1 mV s^−1^. All the curves possessed two peaks: a large one at 4.6–4.8 V caused by the redox of Ni^2+^/Ni^4+^ and a small one at ~4.0 V attributed to the redox reaction of Mn^4+^/Mn^3+^, demonstrating that all the samples possessed an *Fd*3m disordered structure [45], in accordance with the Raman and XPS results. As evident in the magnified images of the Mn^4+^/Mn^3+^ peaks in Figure 7a, LNMO-KCl0.02 and LNMO-KCl0.03 yielded almost the same peak areas as pure LNMO, suggesting that the K^+^/Cl^−^ co-doping did not significantly influence the content of Mn^3+^. However, with increasing K^+^/F^−^ co-doping content, the LNMO-KF0.01 and LNMO-KF0.02 yielded smaller peak areas of Mn^3+^ than the pure LNMO, indicating that K^+^/F^−^ co-doping reduced the Mn^3+^ contents. A low Mn^3+^ ion content may inhibit Jahn–Teller distortion and promote cycling performance [46]. To evaluate the Li^+^ diffusion coefficient, CV tests for the five samples were conducted with a scan rate from 0.1 to 0.5 mV s^−1^, as shown in Figure 7b–f. Table 2 lists the potential differences (∆*V*) between the cathodic and anodic peaks for all the samples. As the scan rate increased, pure LNMO showed the largest potential difference among the five samples. Furthermore, LNMO-KCl0.03 showed the smallest potential difference, indicating that it exhibited the smallest electrode polarization and the best electrochemical reversibility. The lithium-ion diffusion coefficient can be calculated by the following equation:

*I_p_* = (2.69 × 10^5^) *A n*^3/2^ *D_Li_*^1/2^
*C v*^1/2^

where *I_p_* is the peak current, *A* is the electrode area (1.13 cm^2^), *n* is the number of electrons of each molecule in the electronic transfer reaction, *C* is linked to the concentration of Li^+^, and *v* is the scan rate (*v* s^−1^) [25]. Figure 7g,h shows the slope of the line *I_p_* ∼ *v*^1/2^, and the diffusion coefficients of lithium ions for the five samples are given in Table 3. The *D_Li_* for pure LNMO (5.77 × 10^−11^/6.70 × 10^−11^) was smaller than those for LNMO-KCl0.02 (9.08 × 10^−11^/1.06 × 10^−10^), LNMO-KCl0.03 (1.14 × 10^−10^/1.64 × 10^−10^), LNMO-KF0.01 (6.34 × 10^−11^/8.79 × 10^−11^), and LNMO-KF0.02 (8.33 × 10^−11^/7.60 × 10^−11^). The LNMO-KCl0.03 exhibited the largest *D_Li_* because the K^+^/Cl^−^ co-doping expanded the Li^+^ diffusion channels and improved the structural stability. The enhanced *D_Li_* for the LNMO-KF0.02 may have been due to it having the smallest particle size and more positive {100} crystal faces, which shortened the Li^+^ diffusion path and benefitted Li^+^ diffusion. The improved Li^+^ diffusion coefficients for the K^+^/Cl^−^ and K^+^/F^−^ co-doped samples were favorable for enhancing the rate performance.

**Figure 8:**
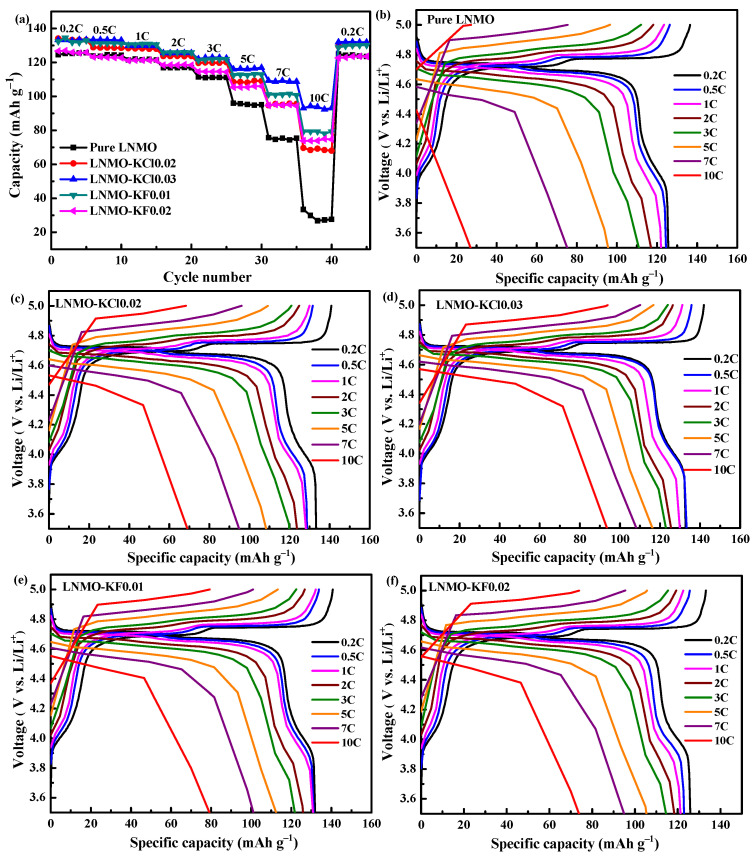
Rate capability of all samples from 0.2C to 10C (**a**); Galvanostatic charge/discharge curves at different C-rates for all samples at 3.5–5.0 V (**b**–**f**).

The rate capabilities of the five electrodes are presented at different C-rates in Figure 8a. K^+^/Cl^−^ and K^+^/F^−^ co-doping effectively enhanced the discharge capacities of the electrode materials compared to those of pure LNMO at high C-rates, as shown in Table 4. Specifically, LNMO-KCl0.03 exhibited the highest discharge capacities of 116.1, 109.3, and 93.9 mAh g^−1^ at high C-rates of 5C, 7C, and 10C, respectively, compared with the values of 95.2, 73.0, and 27.6 mAh g^−1^ for pure LNMO. Figure S6a,b show the rate capabilities for different contents of K^+^/Cl^−^ co-doped LNMO (LNMO-KCl0.02, LNMO-KCl0.03, and LNMO-KCl0.04) and K^+^/F^−^ co-doped LNMO (LNMO-KF0.005, LNMO-KF0.01, LNMO-KF0.02, and LNMO-KF0.03) samples. After an appropriate amount of K^+^/Cl^−^ or K^+^/F^−^ co-doping, the LNMO-KCl0.03 and LNMO-KF0.01 samples delivered optimal rate performances. The rate capability of LNMO-KF0.01 was better than that of the pure LNMO but worse than that of LNMO-KCl0.03. The improved rate capability of LNMO-KCl0.03 was attributed to the synergistic effect of K^+^ and Cl^−^ co-doping, which gives it the highest Li^+^ diffusion coefficient and a stable structure. The charge–discharge curves of all the samples exhibited three plateaus (see Figure 8b–f): the 4.6 and 4.7 V plateaus were assigned to Ni^2+^/Ni^3+^ and Ni^3+^/Ni^4+^ redox reactions, and the 4.0 V platform was ascribed to Mn^3+^/Mn^4+^ redox reactions, corresponding to a disordered *Fd*3m space group, which is in accordance with the Raman and CV results. The relative Mn^3+^ ion contents of the five samples could be qualitatively evaluated by dividing the initial discharge capacity at 0.2C in the range of 3.8–4.3 V [47]. The capacity percentage values decreased to 10.2% for LNMO-KCl0.02, 10.2% for LNMO-KCl0.03, 9.6% for LNMO-KF0.01, and 9.3% for LNMO-KF0.02 compared with 10.4% for the pure LNMO. This result further indicated that the K^+^/Cl^−^ co-doping scarcely affected the Mn^3+^ ion content, while the K^+^/F^−^ co-doping reduced the Mn^3+^ ion content as the K^+^/F^−^ co-doping content increased.

**Figure 9:**
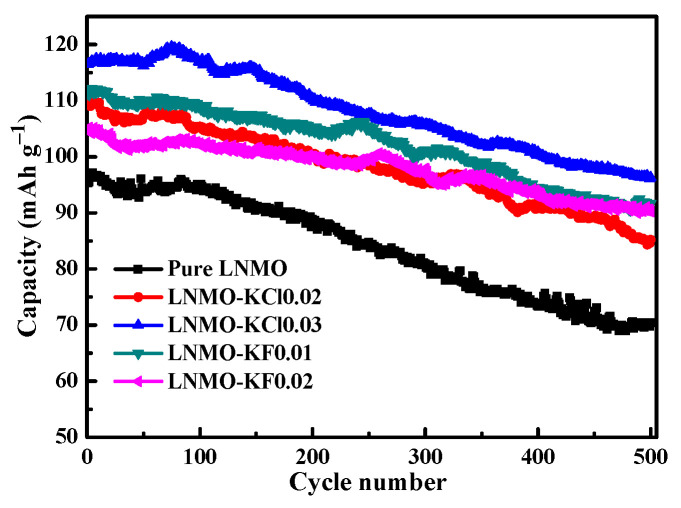
Cycling performances of all samples at 5C for 500 cycles at 3.5–5.0 V.

Figure 9 shows the cycling performances of all the samples, and the data are summarized in Table 5. The pure LNMO delivered low discharge capacity of 70.4 mAh g^−1^ with low capacity retention (73.9%) after 500 cycles at a high rate of 5C. However, increased discharge capacity of 85.2 mAh g^−1^ for LNMO-KCl0.02, 96.3 mAh g^−1^ for LNMO-KCl0.03, 91.7 mAh g^−1^ for LNMO-KF0.01, and 90.2 mAh g^−1^ for LNMO-KF0.02 was observed, corresponding to improved capacity retentions of 77.7%, 82.7%, 82.5%, and 85.8%, respectively. To further understand the cycling behavior, the 300th and 500th charge/discharge curves of the five samples are presented in Figure S7a,b. Compared to pure LNMO, both K^+^/Cl^−^ and K^+^/F^−^ co-doping could alleviate the electrode polarization and improve the electrochemical kinetics. The LNMO-KCl0.03 and LNMO-KF0.02 exhibited the smallest polarization degrees of all the samples. The synergistic effect of the appropriate K^+^/Cl^−^ and K^+^/F^−^ co-doping was responsible for the improved cycling performances of LNMO-KCl0.03 and LNMO-KF0.02. Both K^+^/Cl^−^ and K^+^/F^−^ co-doping reduced the formation of Li*_x_*Ni_1__−_*_x_*O impurities, decreased the particle sizes, expanded the Li^+^ diffusion channels, accelerated the electron migration, and improved the structural stability. Moreover, LNMO-KF0.02 showed the best cycling stability. In addition, the synergistic effect of K^+^ and F^−^ co-doping not only exhibited a truncated octahedral morphology with more {100} faces but also exhibited the smallest particle size and lowest Mn^3+^ ion contents among the five samples. This allowed the Li^+^ diffusion between the active electrode particles to occur more easily, inhibited the Jahn–Teller distortion, and was therefore beneficial to improving the cycling stability.

**Figure 10:**
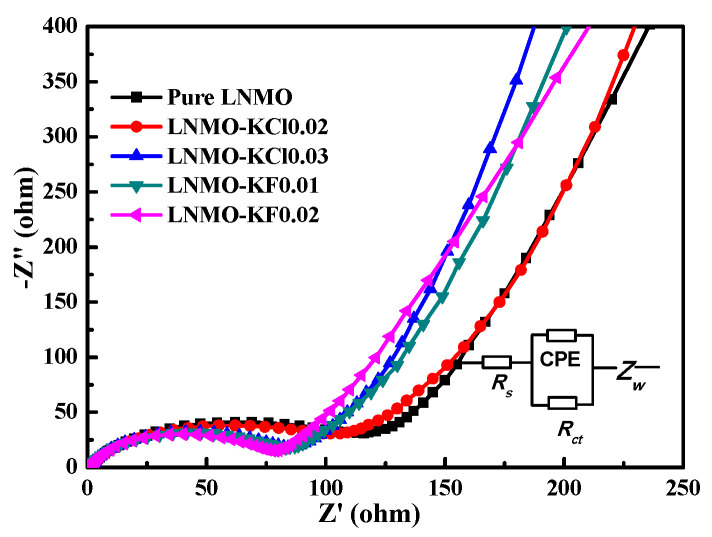
EIS curves (Nyquist plots) and the equivalent circuit (inset) for all samples after 200 cycles at 2C.

To further understand the detailed effects of the K^+^/Cl^−^ and K^+^/F^−^ co-doping on the electrochemical properties of LNMO, EIS plots of the five electrodes after 200 cycles at 2C were obtained, as shown in Figure 10. Consistent with our previous studies [17,25], the five Nyquist profiles were similar. Each was constituted by a single semicircle in the high-to-medium-frequency region and a sloped line in the low-frequency region, which were fitted using the modified Randles–Ershler equivalent circuit shown in the inset of Figure 10. In the equivalent circuit, *R*_s_ represents the electrolyte resistance, *R*_ct_ is the contribution of the charge transfer resistance at the interface of the electrode and electrolyte, and CPE is the double-layer capacitance [48]. The short sloped line is the Warburg impedance (Z_w_), related to the solid diffusion of Li^+^ into the LNMO material. The corresponding fitting results are listed in Table 6. The *R*_ct_ value of pure LNMO (121.8 Ω) was higher than those of LNMO-KCl0.02 (100.5 Ω), LNMO-KCl0.03 (82.6 Ω), LNMO-KF0.01 (81.8 Ω), and LNMO-KF0.02 (77.1 Ω), suggesting that both K^+^/Cl^−^ co-doping and K^+^/F^−^ co-doping could reduce the charge transfer resistance, maintain stable interface structures, and promote charge transfer during the Li^+^ extraction/insertion processes.

## 4. Conclusions

The effects of K^+^/Cl^−^ and K^+^/F^−^ co-doping on the structures, morphologies, and electrochemical performances of LNMO were systematically studied. Both K^+^/Cl^−^ co-doping and K^+^/F^−^ co-doping increased the lattice parameters and reduced the Li*_x_*Ni_1__−_*_x_*O impurities, which expanded the Li^+^ transport channels and improved the structural stability. Furthermore, the K^+^/F^−^ co-doping could also decrease the Mn^3+^ ion content and inhibit Jahn–Teller distortion. Besides this, both the K^+^/Cl^−^ and the K^+^/F^−^ co-doping decreased the particle size and made the particles more uniform, resulting in a shorter Li^+^ ion diffusion distance. Especially at a high C-rate, the LNMO-KCl0.02, LNMO-KCl0.03, LNMO-KF0.01, and LNMO-KF0.02 samples exhibited enhanced rate and cycling performances compared to pure LNMO. The LNMO-KCl0.03 showed the best rate capability with a discharge capacity of 93.9 mAh g^−1^ at 10C and superior cycling performance with capacity retention of 82.7% after 500 cycles at 5C. However, LNMO-KF0.02 delivered the best cycling stability and retained 85.8% of its initial capacity. The excellent electrochemical performances of the K^+^/Cl^−^ and K^+^/F^−^ co-doped LNMO samples will meet the practical application demands of LIBs.

## Figures and Tables

**Table 1:** Lattice parameters of all samples.

Sample	Lattice Parameters		*R*_P_ (%)	*R*_WP_ (%)	Li_x_Ni_1__−__x_O (wt.%)
	*a*/Å	*V*/Å^3^			
Pure LNMO	8.1726(8)	545.8753(5)	10.1	16.0	3.9
LNMO-KCl0.02	8.1771(0)	546.7615(0)	10.4	16.6	3.3
LNMO-KCl0.03	8.1815(2)	547.6486(1)	12.0	17.6	1.5
LNMO-KF0.01	8.1768(6)	546.7133(6)	10.3	16.0	2.4
LNMO-KF0.02	8.1754(0)	546.4205(6)	9.4	14.6	2.0

**Table 2:** Potential differences (∆*V*, V) between anodic (*φ_pa_*, V) and cathodic (*φ_pc_*, V) peaks.

*υ* (mVs^−1^)	Pure LNMO			LNMO-KCl0.02			LNMO-KCl0.03			LNMO-KF0.01			LNMO-KF0.02		
	*φ_pa_*	*φ_pc_*	∆*V*	*φ_pa_*	*φ_pc_*	∆*V*	*φ_pa_*	*φ_pc_*	∆*V*	*φ_pa_*	*φ_pc_*	∆*V*	*φ_pa_*	*φ_pc_*	∆*V*
0.1	4.827	4.624	0.203	4.819	4.608	0.211	4.804	4.624	0.180	4.804	4.623	0.181	4.811	4.617	0.194
0.2	4.849	4.581	0.268	4.852	4.577	0.275	4.836	4.589	0.247	4.838	4.587	0.251	4.842	4.585	0.257
0.3	4.881	4.548	0.333	4.872	4.552	0.320	4.856	4.565	0.291	4.860	4.565	0.295	4.864	4.562	0.302
0.4	4.901	4.519	0.382	4.890	4.534	0.356	4.874	4.547	0.327	4.877	4.545	0.332	4.884	4.541	0.343
0.5	4.921	4.495	0.426	4.907	4.511	0.396	4.893	4.524	0.361	4.890	4.528	0.362	4.90	4.522	0.378

**Table 3:** The diffusion coefficients of Li^+^ in all samples.

Sample	Li-Extraction *D*_Li_ (cm^2^ s^−1^)	Li-Insertion *D*_Li_ (cm^2^ s^−1^)
Pure LNMO	5.77 × 10^−11^	6.70 × 10^−11^
LNMO-KCl0.02	9.08 × 10^−11^	1.06 × 10^−10^
LNMO-KCl0.03	1.14 × 10^−10^	1.64 × 10^−10^
LNMO-KF0.01	6.34 × 10^−11^	8.79 × 10^−11^
LNMO-KF0.02	8.33 × 10^−11^	7.60 × 10^−11^

**Table 4:** Discharge capacity at different C-rates for all samples between 3.5 and 5 V.

Sample	Discharge Capacity (mAh g^−1^)							
	0.2C	0.5C	1C	2C	3C	5C	7C	10C
Pure LNMO	125.7	124.8	121.8	117.0	111.1	95.2	73.0	27.6
LNMO-KCl0.02	133.3	128.7	128.2	123.8	119.8	108.1	95.0	69.2
LNMO-KCl0.03	133.8	133.3	129.9	125.7	123.0	116.1	109.3	93.9
LNMO-KF0.01	131.9	131.2	130.8	125.9	121.8	112.5	101.2	79.3
LNMO-KF0.02	126.9	123.0	121.3	118.4	114.5	105.4	94.9	73.9

**Table 5:** Discharge capacity retention of all samples at 5C after 500 cycles at 25 °C.

Sample	Discharge Capacity at 5C (mAh g^−1^)		Retention at 5C (%)
	1st Cycle	500th Cycle	
Pure LNMO	95.3	70.4	73.9
LNMO-KCl0.02	109.6	85.2	77.7
LNMO-KCl0.03	116.5	96.3	82.7
LNMO-KF0.01	111.2	91.7	82.5
LNMO-KF0.02	105.1	90.2	85.8

**Table 6:** The values of *R*_s_ and *R_ct_* of all samples after 200 cycles at 2C.

Sample	*R_s_*(Ω)	*R_ct_*(Ω)
Pure LNMO	1.6	121.8
LNMO-KCl0.02	1.6	100.5
LNMO-KCl0.03	1.5	82.6
LNMO-KF0.01	1.7	81.8
LNMO-KF0.02	1.7	77.1

## Data Availability

The data presented in this study are available on request from the corresponding author.

## Supplementary Materials

The following are available online at https://www.mdpi.com/article/10.3390/nano11092323/s1, Figure S1: Rietveld refinement results of XRD patterns of pure LNMO (a), LNMO-KCl0.02 (b), LNMO-KCl0.03 (c), LNMO-KF0.01 (d), and LNMO-KF0.02 (e), Figure S2: Rietveld refinement results of XRD patterns of LNMO-KF0.005 (a) and LNMO-KF0.03 (b), Figure S3: SEM images of pure LNMO (a), LNMO-KCl0.02 (b), LNMO-KCl0.03 (c), LNMO-KF0.01 (d), and LNMO-KF0.02 (e), Figure S4: EDS images and the corresponding element mapping of O, Ni, Mn, K, and Cl for LNMO-KCl0.03, Figure S5: EDS images and the corresponding element mapping of O, Ni, Mn, K, and F for LNMO-KF0.02, Figure S6: Rate capability of the samples for pure LNMO, LNMO-KCl0.02, LNMO-KCl0.03, and LNMO-KCl0.04 from 0.2 C to 10 C (a); Rate capability of the samples for pure LNMO, LNMO-KF0.005, LNMO-KF0.01, LNMO-KF0.02, and LNMO-KF0.03 from 0.2 C to 10 C (b), Figure S7: The charge/discharge curves of all the samples in the 300th cycle (a) and 500th cycle (b) at 5C.
