# Supplementary material for: Enhanced Electrochemical Performance of LiNi0.5Mn1.5O4 Composite Cathodes for Lithium-Ion Batteries by Selective Doping of K+/Cl− and K+/F−
Source: Nanomaterials (Basel). 2021 Sep 7;11(9):2323. doi: 10.3390/nano11092323 (PMC8466396; doi:10.3390/nano11092323)
Supplement: Supplementary file 1 [file nanomaterials-11-02323-s001.zip › nanomaterials-1346090-supplementary.pdf]

Supplementary Materials: Enhanced Electrochemical Performance of $LiNi_{0.5}Mn_{1.5}O_4$ Composite Cathodes for Lithium-Ion Batteries by Selective Doping of K^+/Cl^- and K^+/F^-

Aijia Wei [1,2,3], Jinping Mu [1,2], Rui He [2,3], Xue Bai [2,3], Xiaohui Li [2,3], Lihui Zhang [2,3], Yanji Wang [1], Zhenfa Liu [1,2,3,*] and Suning Wang [4]

[1] School of Chemical Engineering and Technology, Hebei University of Technology, Tianjin 300130, China; weiaijia2012@126.com (A.W.); mu18822033042@sina.com (J.M.); yjwang@hebut.edu.cn (Y.W.)

[2] Institute of Energy Resources, Hebei Academy of Sciences, Shijiazhuang, Hebei Province 050081, PR China; kxyherui@163.com (R.H.); bx_ier@yeah.net (X.B.); nys2009@163.com (X.L.); zlhkxy@sohu.com (L.Z.)

[3] Hebei Technology Innovation Center for functional material of lithium battery electrolyte, Shijiazhuang, Hebei Province 050081, PR China.

[4] Guangxi Key Laboratory of Optical and Electronic Materials and Devices, College of Materials Science and Engineering, Guilin University of Technology, Guilin 541004, China; wangsuning0124@outlook.com

* Correspondence: liuzhenfa@hebut.edu.cn

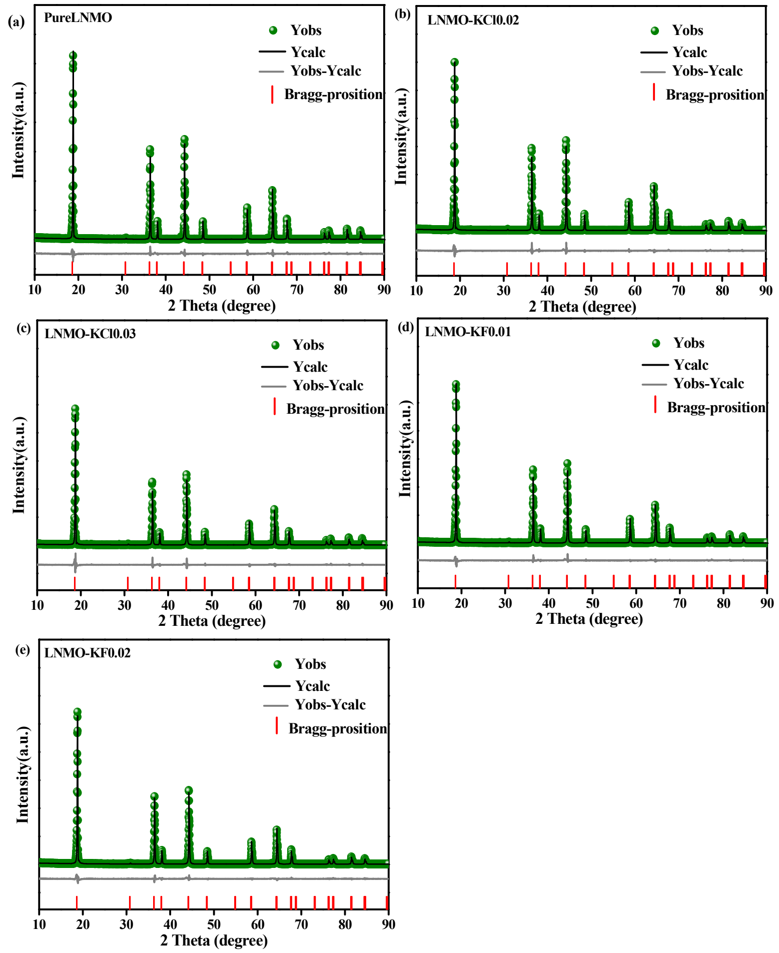

Figure S1. Rietveld refinement results of XRD patterns of pure LNMO (**a**), LNMO-KCl0.02 (**b**), LNMO-KCl0.03 (**c**), LNMO-KF0.01 (**d**), and LNMO-KF0.02 (**e**).

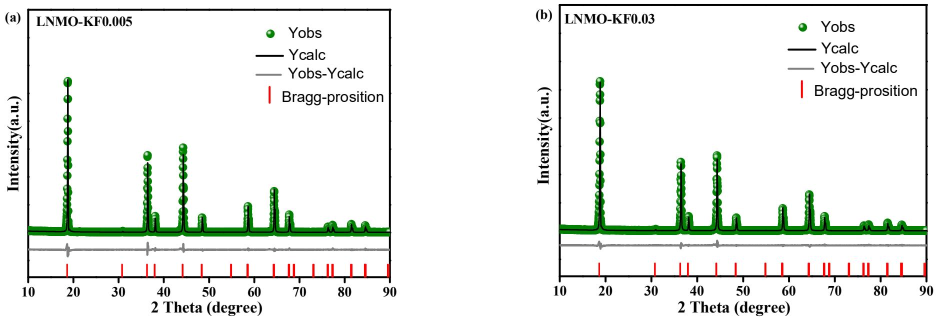

Figure S2. Rietveld refinement results of XRD patterns of LNMO-KF0.005 (**a**) and LNMO-KF0.03 (**b**).

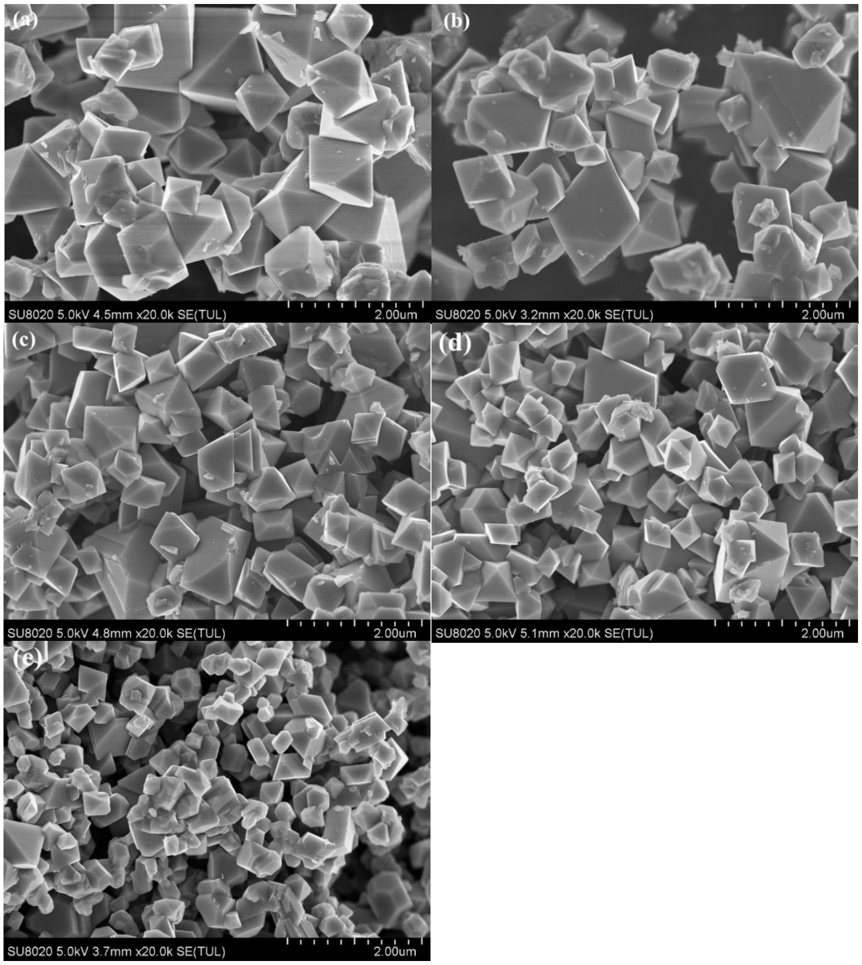

Figure S3. SEM images of pure LNMO (**a**), LNMO-KCl0.02 (**b**), LNMO-KCl0.03 (**c**), LNMO-KF0.01 (**d**), and LNMO-KF0.02 (**e**).

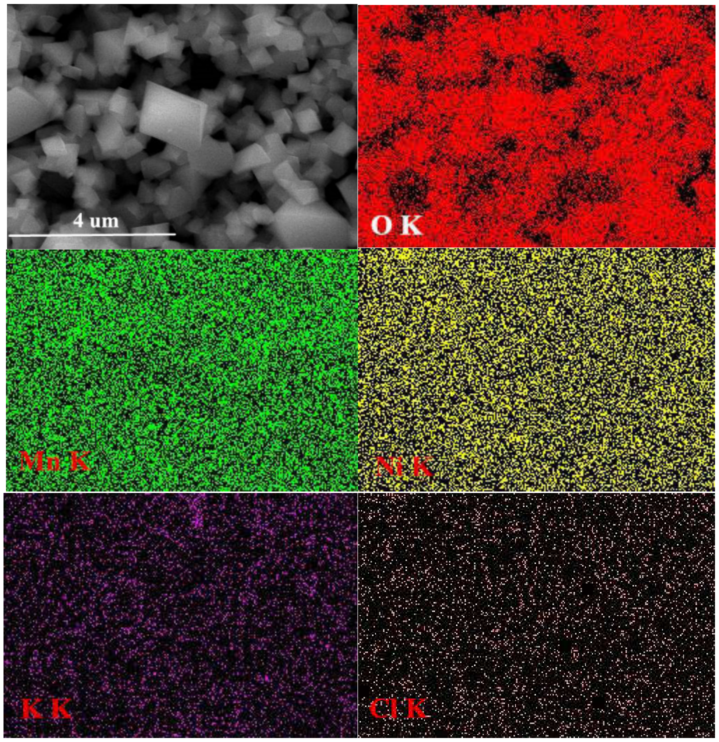

Figure S4. EDS images and the corresponding element mapping of O, Ni, Mn, K, and Cl for LNMO-KCl0.03.

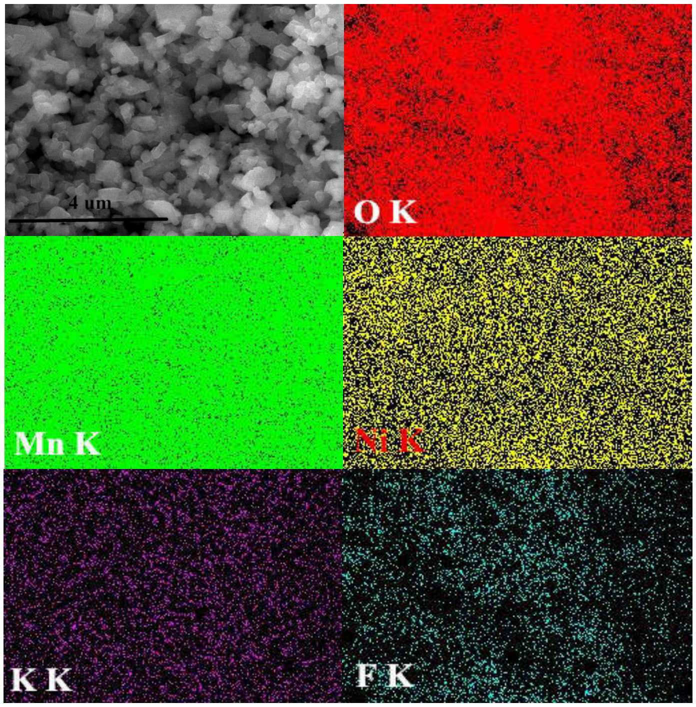

Figure S5. EDS images and the corresponding element mapping of O, Ni, Mn, K, and F for LNMO-KF0.02.

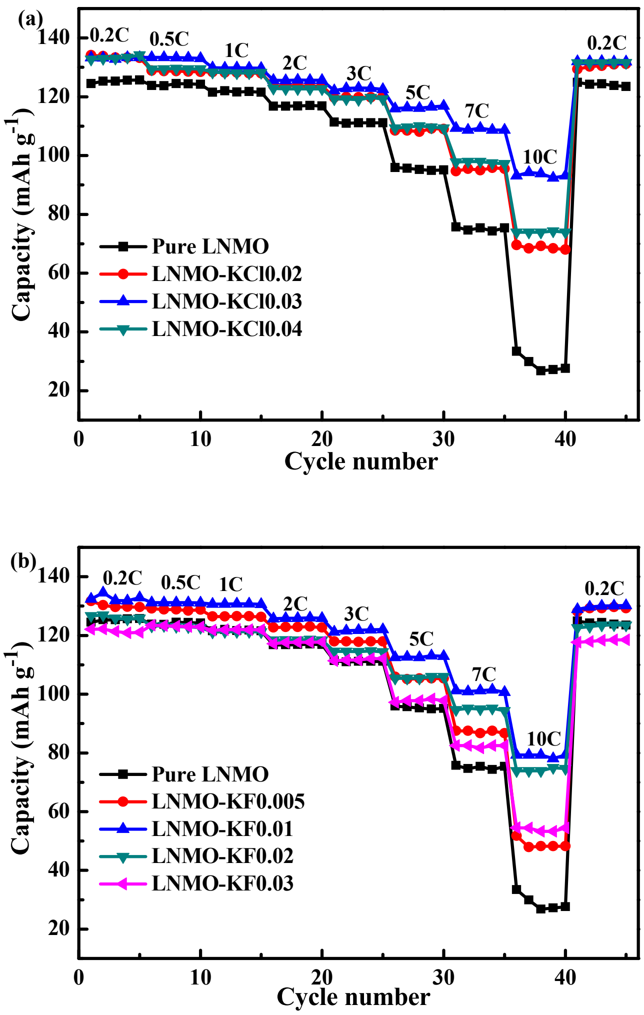

Figure S6. Rate capability of the samples for pure LNMO, LNMO-KCl0.02, LNMO-KCl0.03, and LNMO-KCl0.04 from 0.2 C to 10 C (**a**); Rate capability of the samples for pure LNMO, LNMO-KF0.005, LNMO-KF0.01, LNMO-KF0.02, and LNMO-KF0.03 from 0.2 C to 10 C (**b**).

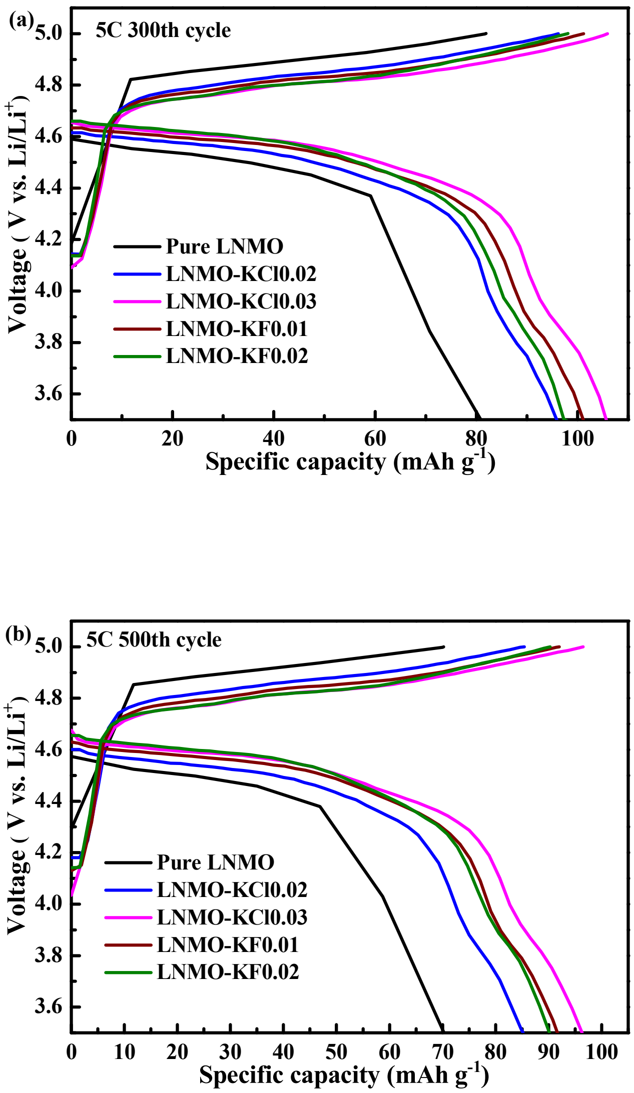

Figure S7. The charge/discharge curves of all the samples in the 300th cycle (**a**) and 500th cycle (**b**) at 5C.
